# Can Scabies Be Cured by the Simillimum?

**Published:** 1893-03

**Authors:** E. T. Adams

**Affiliations:** Toronto, Can.


### CAN SCABIES BE CURED WITH THE SIMILLIMUM?

E. T. Adams, M. D., Toronto, Can.

Mr. President and Brethren:—To my mind there are two important matters affecting our "Common Israel" very closely which demand and require settlement, or as near a settlement as our present knowledge and experience will permit.

Wonderful is the advance made in the study and practice of pure Homœopathy in the past eleven years, or since a few of our noble seniors came out from among the majority and decided that they would no longer be placed in a position where they could be accused of hypocrisy, etc.

I should have said wonderful is the return to the study and practice of the fathers. I sometime doubt whether we, who from age or lack of knowledge of the truth, were not among that noble band appreciate what they then and there did for us, and when in sorrow I study o'er and think of the blind, egotistic ignorance in which I then labored and practiced, I honestly and with a thankful heart bless those fathers, and with a heart-felt gratitude bless the day when they unfurled again to the world the flag of pure Homœopathy, and regard that day as only second to that on which Hahnemann—THE MASTER—first announced to the world the blessing of Homœopathy and its talisman, "*Similia simillibus curantur.*"

To get back to business, and my first matter or question, whatever the result, I beg you will consider and believe that my intention is good and for the advancement of the truth.

In our meetings and reports we often read of "spoiled cases," "that no hope of cure remains," etc., etc.

I have long felt that the truth of such assertions should be definitely ascertained before permitting their use; in fact, that they should be axiomatic to excuse or permit of their use at all.

If not true then such expressions have much to answer for, and if true, then we have much to answer for.

In my journey from darkness to light, from ignorant eclecticism to some knowledge of the truth as it is in Hahnemann, I may honestly say that I found this expression of a belief in this possibility to be my greatest obstacle—the chief stumbling-block in my upward journey. My desire in bringing this question before this Association is to elicit a discussion in which we may obtain the truth as found in the experience and practice of many here, who have greatly aided in the past in the settlement of debatable questions which have occurred in our medical system. Briefly, then, let me ask why and how it is possible that we, with the minimum dose, repeated infrequently, can produce such *fatal* results—fatal is the only appropriate term—that the victim may give, nay, must give up all hope of cure, while prior to the administration of this medicine in error the condition of the patient was quite curable? Truly, as we are but finite men, if it be proven that such sad effects may follow a mistake of judgment, work, or lack of ability, over our office-doors should be written, "All ye who enter here may leave all hope behind." The vast majority of our bad chronic cases come from allopathic hands whence they have received innumerable and strong doses of powerful drugs and poisons, yet, I think, with the possible exception of cases of heart disease treated by massive doses of Digitalis (and even the truth of this is not invariably admitted), we do not regard such patients as being hopelessly incurable, but get to work, and with the indicated remedy cure the majority.

To my mind, if forced to accept such belief as true, we take upon our shoulders, to say nothing of our consciences, a burden almost unbearable.

Then, again, let me ask why a remedy selected through careful study and conscientious comparisons should produce such disastrous results? I think you will admit that a remedy so selected is, if not the simillimum, at least a similar. If this be granted I will remind you that the fathers of our school, in the days antecedent to ours, with our enlarged and improved works on Materia Medica (*improved* is not ironical, as I do not refer to *all* modern works) and before the introduction and proving of scores of "new remedies" made wonderful cures, established the truth of our law by the use of such similars, zigzagging back and forth with their similars until health was restored and cures effected. If the danger of "spoiled cases" exists at all surely they should be more common in those days and under those more trying conditions.

I may add that my remarks also refer to like results when said to come from a too frequently or too early repeated dose. To my mind the punishment is too great for the offense.

I cannot imagine a Hahnemannian physician trying faithfully to select the simillimum and using his best judgment as to the repetition of the dose of the simillimum who merits such severe punishment from an unintentional error.

You have the question before you. For long years it has worried and troubled me, lest with my best efforts to benefit an unfortunate sufferer I might unintentionally blast his future.

It is not to cavil, but with anxiety for relief and knowledge, I introduce this question.

My second question is on the treatment of pure scabies. From personal knowledge and experience I know that among us exists the greatest dissatisfaction as to the results obtained from the treatment of this disease, and this among men who practice Hahnemannian Homœopathy and successfully in all other curable affections, yet with their best efforts, exercised with the greatest care, they make failures right along, and after months of treatment only find the patient growing worse and worse, till the friends, becoming not only discouraged but disgusted, call in old school aid, when lo! presto! in a few days the scourge of months is conquered and the prisoner liberated—and by the use of external remedies.

I feel strongly on this question, for I have suffered, and in my efforts to practice purely have seen families leave me, and, what is infinitely worse, leave Homœopathy.

Is it not rather strange that men who have good success in the treatment of other affections should fail so egregiously in this disease? There must be a cause—a reason—why physicians who cure the whole list of itching, irritative eruptions—including herpes, eczema, purigo, etc., etc., should fail entirely over scabies. Truly in this case the question of "what's in a name" is important. I defer to none in the reverence and honor with which I regard Hahnemann, but many and many a time, when disheartened with the lack of success after months of careful treatment of such cases, I have wondered whether Hahnemann did not regard all itching eruptions as from the same root and designate the whole as "itch"—and it is not always an easy task to correctly diagnose between the pure and spurious—so much is this the case that I have come to regard a very few cases I have cured in the past eight or nine years, as being spurious and not the acarus scabei. Here, I would like to ask what injury would be liable to result to the patient, who while under constitutional treatment, used Sulphur ointment as a germicide?

It will be said, I doubt not, that strict adherence to our law, the selection of the simillimum from the totallity of symptoms will cure pure scabies or acarus scabei, and I doubt not honestly, but there comes the question asked before, why other Hahnemannians, successful above the average, should fail with this diseased condition so disastrously ?

This question is agitating our men "across the line," and that I am not alone I have here in brief form the experience of one of our men, a Hahnemannian from principle, a student of our greatest teacher of the philosophy of Homœopathy and materia medica, and a young man who devotes much time and great care to the study of his cases. Also, a brief article from one of our older members entitled the "Sphere of Homœopathy," which, as you will hear, is appropriate to this question.

